# Annals of Thoracic Medicine … a three-year journey

**DOI:** 10.4103/1817-1737.44776

**Published:** 2009

**Authors:** Mohamed S. Al-Moamary

**Affiliations:** *Clinical Affairs College of Medicine, King Saud bin Abdulaziz University for Health Siences, Riyadh, Saudi Arabia*

There is a paucity of published scientific data from the Middle East region in the fields of respiratory and critical care medicine. There is also limited information regarding the types of respiratory diseases prevalent in this region.[1] This is despite the fact that a large number of qualified respiratory physicians are conducting research in the Middle East, albeit with limited resources. In recent years, the trend has been toward supporting research by assigning resources in the form of facilitating grants and by founding scientific societies that promote research. In keeping with this, state-of-the-art research centers are being established in the gulf region, and it is expected that more journals will be needed in the near future to accommodate the large amount of research data that will be generated by these centers. In November 2008, one speaker at the conference of the Eastern Mediterranean Association for Medical Editors in Bahrain pointed out that 408 journal were registered in the region; 57% of these journals are active and 184 are available online.[2]

**Figure 1:**
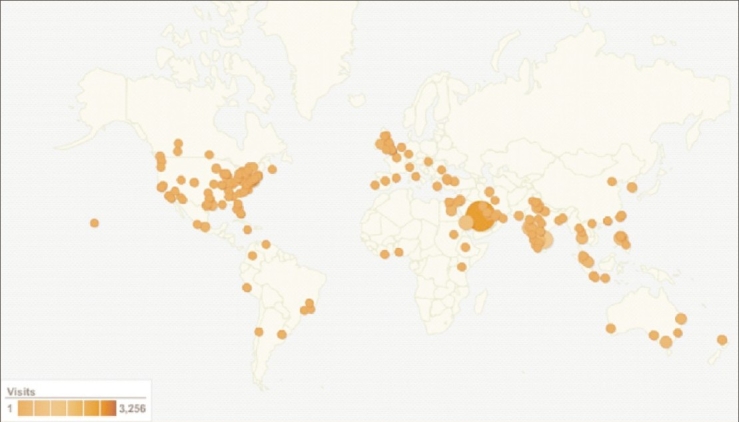
Worldwide distribution of the readers of Annals of Thoracic Medicine

The Annals of Thoracic Medicine (ATM) (www.thoracicmdicine.org) was founded by The Saudi Thoracic Society to allow original research, experience, and ideas to be communicated to the national and international scientific community. The journal was also expected to encourage local physicians to conduct and publish clinical and basic research related to local diseases. The ATM has also taken the initiative to publish locally applicable guidelines for the management of diseases such as pulmonary hypertension, lung cancer, and bronchial asthma. ATM has often invited national and international experts to write articles on the latest developments in the fields of respiratory and critical care medicine. Furthermore, the journal strives to adhere strictly to international standards and ensures that each issue is released on time. There is also emphasis on the ethics of research so as to avoid research misconduct. All manuscripts are initially screened with a computer software to detect fraud and plagiarism. The availability of ATM on the Web as an open access has made the journal accessible from all over the world [Figure 1]. ATM allows researchers from different countries to submit their papers by accessing the online manuscript-management system (www.journalonweb.com/atm). The number of articles submitted to ATM has increased over the last 3 years [Table 1]. As part of the effort to support quality research, the editorial board has successfully reduced the time from submission of an article to acceptance to an average of 38 days for the year 2008. Almost two-thirds of the submissions come from outside Saudi Arabia. Over the last 3 years, ATM has been indexed in more than 20 search engines as well as in databases such as Science Citation Index. The editorial board of ATM aims to increase the visibility and readability of the journal and indexing in all major search engines and databases. Over the last 3 years, ATM has continued to adhere to stringent international standards and will remain a reputable and leading journal in respiratory and critical care medicine and other related disciplines in the Middle East.[3]

**Table 1:** Annual data from the Annals of Thoracic Medicine since it was established in the year 2006

Year	Submission acceptance time (in days)	Rejection rate (%)	Percentage of manuscripts from abroad (%)
2006	44	36	62
2007	48	56	74
2008	38	62	62
